# Bioaccessibility of Plant Sterols in Wholemeal Rye Bread Using the INFOGEST Protocol: Influence of Oral Phase and Enzymes of Lipid Metabolism

**DOI:** 10.1021/acs.jafc.2c04024

**Published:** 2022-10-07

**Authors:** Nerea Faubel, Mussa Makran, Antonio Cilla, Amparo Alegría, Reyes Barberá, Guadalupe Garcia-Llatas

**Affiliations:** Nutrition and Food Science Area, Faculty of Pharmacy, University of Valencia, Av. Vicente Andrés Estellés s/n, 46100 Burjassot, Spain

**Keywords:** cholesterol esterase, gastric lipase, human chewing, *in vitro* digestion, solid matrix, phytosterols

## Abstract

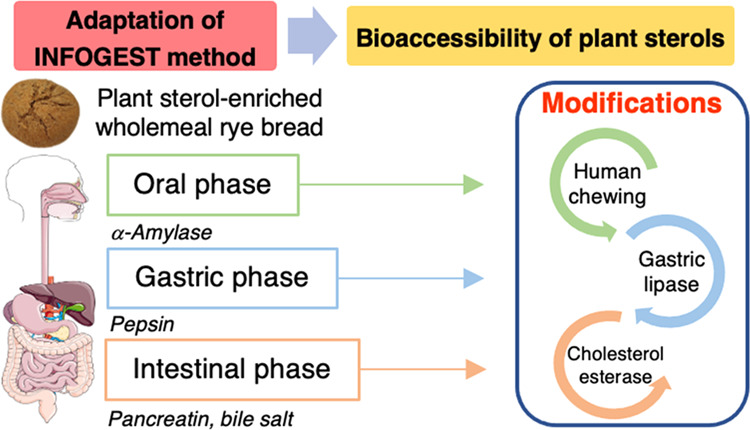

Bioaccessibility of plant sterols (PS) in an enriched wholemeal rye bread was evaluated, for the first time, using the INFOGEST protocol without gastric lipase (GL) and cholesterol esterase (CE), with GL or GL + CE. Moreover, human chewing and an *in vitro* oral phase (simulated salivary fluid and α-amylase) were evaluated for this purpose. The addition of GL decreased the bioaccessibility of total PS (from 23.8 to 18.5%), whereas the use of GL + CE does not significantly affect PS bioaccessibility. The *in vitro* oral phase resulted in an ineffective homogenization of the fresh *vs* partially dried and milled bread, reducing the bioaccessibility of total (from 20.2 to 12.8%) and individual PS. The INFOGEST digestion including the use of GL and CE, as well as an oral phase with human chewing, is proposed for the assessment of PS bioaccessibility in a solid matrix such as wholemeal rye bread since it more closely approximates the *in vivo* situation.

## Introduction

Daily intakes of 1.5–3 g of plant sterols (PS) have shown to be effective cholesterol-lowering agents, decreasing plasma concentrations up to 12%.^1^ Other biological actions of PS are antiproliferative, anti-inflammatory, antioxidant, and antidiabetic.^2^ Minor side effects with PS have been reported, including nausea, indigestion, diarrhea, flatulence, and others,^3^ and atherogenic effects only in sitosterolemic individuals.^4^ To achieve PS effective intakes, the European Union has allowed PS fortification and commercialization of certain foods such as rye bread.^5,6^

Wholemeal rye bread (WRB) is an excellent source of fiber (arabinoxylan, fructan, cellulose, and β-glucan),^7^ which is related to cardiovascular protection. It has been proven a decrease in serum total and LDL cholesterol in an *in vivo* study of healthy, free-living, normocholesterolemic individuals consuming rye bread (99 g) enriched with 2 g of PS every day for two weeks and then doubling the intake (198 g of bread providing 4 g of PS) every day during another two weeks.^8^

From a nutritional and functional point, it is crucial to know not only just the amount of lipophilic bioactive compounds such as PS in foods but also their bioavailability. For this purpose, *in vivo* methods offer the most reliable results and are used as reference standards, however, they have several drawbacks, such as high equipment costs, ethical restraints, lengthy processes, and high variability.^9,10^ Hence, *in vitro* approaches are an excellent tool for assessing the bioaccessibility of compounds (soluble fraction to be possibly absorbable in the intestinal phase) as a screening method prior to *in vivo* models.^11^ Thus, *in vitro* gastrointestinal digestion methods are used, such as the static INFOGEST model, a standardized simulation developed by the COST Action INFOGEST network that has been developed to mimic *in vivo* conditions of digestion.^12^ Egger et al.^13^ have demonstrated the suitability of this protocol for evaluating protein hydrolysis in skim milk powder due to its similarity with *in vivo* pig digestion.

In the case of lipophilic compounds, such as PS, the presence of enzymes of lipid metabolism like gastric lipase (GL) and cholesterol esterase (CE) in digestion is important, as their participation has been demonstrated in *in vivo* digestion.^14^ The INFOGEST method has been updated by incorporating GL.^15^ It has been shown that GL acts by hydrolyzing triacylglycerides, which leads to an increase in free fatty acids (FFA) and monoacylglycerides (MAG). These emulsifying agents produced by lipolysis promote the micellarization of lipophilic compounds, such as PS, by means of a better incorporation into the mixed micelles,^14,16^ which turns into a higher bioaccessibility. However, bile salts used as a digestion reagent contain cholesterol preformed in micelles, which is preferentially incorporated into the mixed micelles, thus hindering the incorporation of PS.^17^ Although CE is not included in the INFOGEST model, it is also a key enzyme in lipid metabolism, being able to hydrolyze esterified sterols and triacylglycerides, promoting sterol micellarization as well as acting as a supplementary enzyme in lipolysis.^18^ A semidynamic method beholding a dynamic gastric phase together with the oral and intestinal phases of the INFOGEST method applicable for the determination of digestibility of nutrients has been proposed by Mulet-Cabero et al.^19^ as a new adaptation of the INFOGEST model.

In the case of solid foods, the oral phase has a crucial effect on their disruption and, thus, on the digestibility and solubility of nutrients and bioactive compounds. In this regard, the influence of different parameters of an *in vitro* oral and gastric digestion (non-INFOGEST) was evaluated to determine the rate of bread breakdown during digestion in different types of bread (white, wheat, rye, barley, and almond-wheat).^20^ Furthermore, an oral phase with human chewing for WRB,^21^ wholemeal wheat bread (WWB),^21−23^ and white bread^23−25^ has been evaluated, although for assessing parameters of the oral phase. According to the INFOGEST protocol^15^ for solid and starchy foods, an oral phase including α-amylase must be carried out.

The bioaccessibility of steryl ferulates in cargo rice, rice bran, corn bran, and wheat bran including an oral phase with artificial saliva mixed with α-amylase followed by gastrointestinal digestion with CE provided by pancreatin but without GL has been evaluated.^26^ Subsequently, these authors^27^ digested breads with integral wheat flour and combined with white wheat flour and evaluated the bioaccessibility of steryl ferulates by applying the same digestion conditions. Moreover, the bioaccessibility of total phytosterols has been evaluated in PS-enriched oat granola bars formulated with different amounts of fat (24, 7, and 0 g lipid/100 g). The digestion of the grounded granola bars included an oral phase with simulated salivary fluid (SSF) and α-amylase, followed by the gastric phase with the addition of fungal lipase (nongastric) and the intestinal phase without CE.^28^ A recent study^29^ has evaluated the behavior of human chewing and the digestibility of protein and starch after a modified INFOGEST method in black beans.

In a previous study of our research group,^30^ the effect of the addition of GL and/or CE in the INFOGEST digestion model for assessing the bioaccessibility of PS in a liquid matrix (PS-enriched milk-based fruit juice beverage) has been evaluated. However, no studies have determined the bioaccessibility of PS in enriched or nonenriched rye bread. Therefore, the aim of this study is to evaluate, for the first time, the influence of different oral phase conditions (human chewing *vs in vitro* oral phase) on the bioaccessibility of PS in a solid food matrix, such as a 100% WRB enriched in these compounds by applying the INFOGEST method as a basis. Moreover, the effect of the addition of key enzymes of lipid metabolism (GL and CE) on the bioaccessibility of PS in the WRB has been studied in this work as a novelty.

## Materials and Methods

### Chemicals and Reagents

The sterol standards used were 5β-cholestan-3α-ol (epicoprostanol) (purity 99%) as an internal standard (IS), 5,22-cholestadien-24-ethyl-3β-ol (stigmasterol) (purity 97%), and 24α-ethyl-5α-cholestan-3β-ol (sitostanol) (purity 97%) purchased from Merck Life Science S.L.U. (Madrid, Spain). 24α-Methyl-5-cholesten-3β-ol (campesterol) (purity 98%) and 5-cholesten-24β-ethyl-3β-ol (β-sitosterol) (purity 98%) were acquired from Chengdu Biopurify Phytochemicals Ltd (Sichuan, China). Derivatization reagents anhydrous pyridine and trimethylchlorosilane (TMCS) were obtained from Acros Organics (Geel, Belgium), and hexamethyldisilazane (HMDS) was acquired from Merck Life Science S.L.U. (Madrid, Spain). α-Amylase from human saliva (E.C 3.2.1.1), ammonium carbonate, ammonium chloride, anhydrous sodium sulfate, bovine bile, calcium chloride dihydrate, CE from porcine pancreas (E.C 3.1.1.13), hydrochloric acid (purity 37%), magnesium chloride hexahydrate, pancreatin from porcine pancreas, pepsin from porcine gastric mucosa (E.C 3.4.23.1), potassium chloride, potassium dihydrogen phosphate, potassium hydroxide, sodium chloride, and sodium hydroxide were purchased from Merck Life Science S.L.U. (Madrid, Spain). Rabbit gastric extract (RGE) was acquired from Lipolytech (Marseille, France). Ethanol, sodium bicarbonate, and sodium hydroxide were obtained from Panreac (Barcelona, Spain). Cyclohexane, diethyl ether, and hexane were supplied by Scharlau (Barcelona, Spain). A Milli-Q system was used to purify water (Milford, MA). The reagents used for enzymatic activity assays have been described elsewhere.^30^

### Sample Preparation

The ingredients (w/w of dough) used for the bread-making procedure of the PS-enriched 100% WRB were whole rye flour (57%), compressed yeast (1.4%), common salt (0.9%), ultrapure water (38.2%), ascorbic acid (0.006%), and an ingredient containing microencapsulated free PS from tall oil (2.5%) (Lypophytol 146 ME Dispersible, Lipofoods, Barcelona, Spain). The WRB production method began with combining all of the materials in a spinning blade mixer. The bread dough was then rested for 10 min before being split into different pieces. These were handballed and set aside for another 15 min to rest. After that, the dough was fermented for 45 min at a temperature of 28 °C and relative humidity of 85%. The bread doughs were fermented before being cooked for 25 min at 180 °C. The weight of the individual loaves was 81.3 ± 1.1 g, providing 1.8 g of PS in each loaf. The nutritional composition determined for the WRB (g/100 g) was as follows: lipids, 3.2 ± 0.1; ash, 1.37 ± 0.04; proteins, 5.16 ± 0.03 (*f* = 5.83); insoluble fiber, 10.3 ± 1.4; soluble fiber, 3.4 ± 0.1; and carbohydrates, 43.9 ± 0.4.

In this work, the WRB was used fresh or partially dried and milled. To obtain partially dried and milled WRB, it was placed in an oven at 24 °C overnight to preserve microbiological quality and grounded with a kitchen mincer.

### Human Oral Phase Preliminary Studies

#### Subjects

Six volunteers (four males and two females, age range: 22–42 years) had participated in the study. Inclusion factors were healthy and complete dentition, free from functional mastication problems and no dental treatment in the three months before experimentation, and no medication that might influence mastication.

#### Food Oral Processing of the Sample

The assayed oral processing has been made according to Assad-Bustillos et al.^31,32^ and Aleixandre et al.^23^

All subjects gave their informed consent to participate in the study and were asked not to eat or drink for at least one hour before the session. Portions of 5 g of fresh WRB (5.2 ± 0.1) were cut just before the beginning of the experimentation, with the same proportion of crumb and crust, and offered to the participants. They were asked to consume the sample mouthful in a natural manner as they do at home. The WRB portion was placed in the mouth by the subjects who were instructed to close their mouth before starting to chew. The participants, on a signal from the instructor, began to chew and time started to count down. Then, they had to raise their hand when they wanted to swallow the bolus and at that moment the time stopped counting down. The first sample had to be chewed and expectorated just before the subjects felt the need to swallow and discarded to familiarize them with every step of WRB chewing. Subsequently, participants were asked to chew the next three samples mouthful and to expectorate the food bolus into a preweighed plastic sterile bottle after complete mastication.

Total chewing duration was calculated as the time between the first chew and the swallowing time (s), which was recorded by a digital chronometer. The number of chewing cycles was also determined from this recording, and one chewing cycle was defined as a complete sequence of opening and closing movements of the maxilla. Chewing frequency was calculated by dividing the number of chewing cycles by the chewing duration experimental procedure. The amount of saliva incorporated into the expectorate bolus was calculated as the difference between the weight of the bolus and the weight of the bread sample, and the sample/saliva ratio was calculated.

#### Determination of Human α-Amylase Activity

The collection of saliva was carried out according to Sahu et al.^33^ The subject was not allowed to eat or drink for two hours before the collection, except water. The saliva accumulated in the mouth cavity throughout 10 min was collected in a preweighed plastic vial, and it was weighed every 2 min to estimate the saliva flow rate. Then, the saliva sample was centrifuged at 10,000*g*, and the supernatant was used for determining the α-amylase activity.

An α-amylase activity is required between 1 and 3 U/mL in saliva, according to the protocol for its determination.^15^ Therefore, taking into account that the average α-amylase activity for men was 160 U/mL saliva,^33^ a dilution of saliva between 1/100 and 1/200 was considered optimal. The enzyme activity was determined according to the protocol indicated in the INFOGEST model.^15^ Unit definition: one unit releases 1.0 mg of maltose equivalent from starch in 3 min at pH 6.9 and 20 °C.

### Oral–Gastrointestinal Simulated Digestion

#### Determination of Enzyme Activity

The activities of commercial human α-amylase, porcine pepsin, RGE, and pancreatin from porcine pancreas and the bovine bile salt content were experimentally determined in two independent assays (at least *n* = 3 per assay), as indicated in a previous study,^30^ according to INFOGEST procedures.^15^ Since the RGE contains both GL and pepsin, the pepsin activity was also determined in the RGE to recalculate the amount of porcine pepsin to be added in the digestion. The CE activity of the batch corresponds to that provided by the manufacturer since the use of CE is not contemplated in the INFOGEST guideline protocol and no defined methodology for assessing its activity is stated.

#### Digestion Conditions Assayed

**Figure 1:**
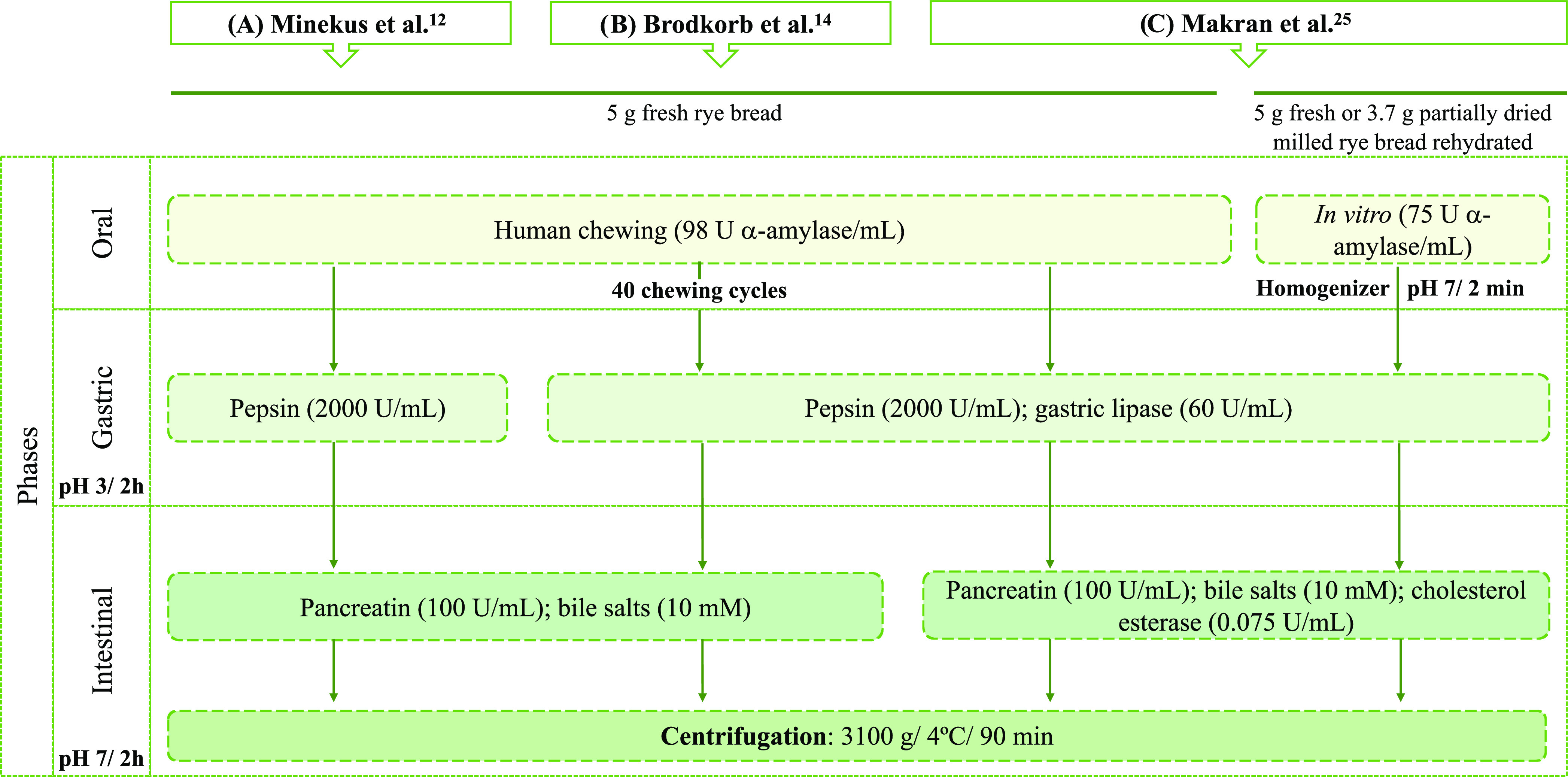
Schematic representation of the *in vitro* gastrointestinal digestion conditions assayed in this study.

Three methods were used: (A) the standardized *in vitro* static digestion model INFOGEST reported by Minekus et al.^12^ as the basis for this study; (B) the update INFOGEST 2.0 protocol described by Brodkorb et al.^15^ with the addition of GL at 60 U/mL; and (C) the method applied by Makran et al.^30^ adding GL at 60 U/mL and CE at 0.075 U/mL. Human oral phase was used in digestion methods A, B, and C, and *in vitro* oral phase was used only in method C (Figure 1).

For the human oral phase, 5 g of fresh WRB was chewed as aforementioned in the “Food Oral Processing of the Sample” section. For the *in vitro* oral phase (only for method C), 5 g of fresh or 3.7 g of partially dried and milled WRB rehydrated with ultrapure water, according to humidity bread (26.2%), was mixed with 3.5 mL of SSF and shaken for one min in a homogenizing sample (Masticator Basic 400, IUL, Barcelona, Spain). Then, the α-amylase solution at 1500 U/mL (0.5 mL) was included to achieve a final concentration of 75 U/mL in the oral digesta. Calcium chloride at 0.3 M (25 μL) was added, and the pH was adjusted up to 7 and completed to a volume of 10 mL with water. The mixtures were shaken for 2 min at 37 °C and 95 rpm in a shaker water bath (SBS40 Stuart, Staffordshire, U.K.).

In the gastric phase, simulated gastric fluid (SGF) (7.5 mL), pepsin solution at 25,000 U/mL (1.6 mL) for a final concentration of 2000 U/mL in the gastric digesta, and 0.3 M calcium chloride (5 μL) were added and manually mixed for one min. For methods B and C, GL was included in the digestion from an RGE solution at 225 U/mL (0.98 mL) for a final concentration of 60 U/mL in the gastric digesta. Since the RGE also provides pepsin activity, the pepsin solution at 25,000 U/mL (0.62 mL) was added to achieve a final concentration of 2000 U/mL in the gastric digesta. The pH of the mixture was adjusted to 3 and completed to 20 mL with water. The gastric mixture was placed in a shaker bath for 2 h at 37 °C and 95 rpm.

For the intestinal conditions, simulated intestinal fluid (SIF) (11 mL), pancreatin solution at 800 U/mL based on trypsin activity (5 mL) for a final concentration of 100 U/mL in the intestinal digesta, 0.3 M calcium chloride (40 μL), and bovine bile extract solution at 166 mM (2.5 mL) for a final concentration of 10 mM in the intestinal digesta were added. For method C (GL + CE), CE was incorporated (0.1 mL of the 30 U/mL CE solution to obtain an activity of 0.075 U/mL). The resulting mixture was manually shaken for one min, and the pH was adjusted to 7 and completed to 40 mL with water. Finally, the digesta was shaken for 2 h at 37 °C and 95 rpm in a shaking water bath. To obtain the supernatant, which corresponds to the bioaccessible fraction (BF), the digesta was centrifuged (Eppendorf centrifuge 5810R, Hamburg, Germany) at 3100*g*, 4 °C, and 90 min.

Digestions were carried out in triplicate. The respective blanks of digestion for each of the different digestion conditions (A, B, C) were carried out, in the same way as the samples, to subtract the sterol content in the BF from the digestion reagents. Sterol bioaccessibility was estimated as a percentage of sterols present in the BF compared to those present in the WRB (undigested) as follows
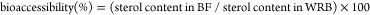


### Determination of Sterols

The methodology used for the determination of sterols in the WRB and the BF was according to Piironen et al.^34^ with slight modifications. Briefly, 0.35 g of partially dried and milled WRB was mixed with IS (200 μg) and 1 mL of absolute ethanol and subjected to acid hydrolysis with HCl at 80 °C for 1 h. The fat was extracted with hexane/diethyl ether (1:1, v/v) and centrifuged at 500 rpm for 10 min at room temperature by repeating this procedure twice. The collected organic phases were evaporated to dryness with a rotary evaporator (50 °C) (Rotavapor R-200 with a heating bath B490, Büchi, Flawil, Switzerland) and dissolved in absolute ethanol. Hot saponification was applied to the BF (2 mL was mixed with 200 μg of IS and 1 mL of absolute ethanol) and the fat extracted from WRB. Saturated aqueous KOH was added, and samples were heated at 80 °C for 30 min in a shaker water bath at 100 rpm. The extraction of the unsaponifiable fraction was done with water and cyclohexane, and samples were subsequently shaken. Then, the organic phase was evaporated to dryness with a rotary evaporator (50 °C) and dissolved with hexane.

SPE (solid-phase extraction) with silica cartridges (Finisterre SPE tube Si, 500 mg/6 mL, Teknokroma, Barcelona, Spain) was used for purification of the organic extract, and sterols were eluted with hexane/diethyl ether (1:1, v/v). The solvent was removed under a stream of nitrogen, and the residue was dissolved in hexane. Derivatization was carried out with pyridine/HMDS/TMCS (5:2:1, v/v/v) at 40 °C during 25 min (SBH200D Blockheater, Stuart, Staffordshire, United Kingdom).^35^ Then, the reagent was evaporated, and the trimethylsilylether derivatives were dissolved in hexane and filtered (syringe-driven Millex FH with a filter 1 mL, 0.45 μm, Millipore, Milford, MA). The solvent was again evaporated, dissolved in 100 μL of hexane, and analyzed (1 μL) by gas chromatography-flame ionization detector (GC-FID) (YL Instrument 6500 GC System, Gyeonggi-do, Korea) equipped with a capillary column (CP-Sil 8 low bleed/MS 50 m × 0.25 mm × 0.25 μm film thickness), according to the conditions detailed elsewhere.^30^ Sterols were identified by comparing their relative retention times with those of the standards derivatized by the same procedure as the samples and on the basis of the elution pattern indicated elsewhere.^30,34,36^ The quantification was performed using calibration curves developed with the sterol standards (Table 1). Sitostanol curves were used to quantify sitostanol and campestanol, and the β-sitosterol curve of lower quantity ranges was used to quantify Δ5-avenasterol, Δ5,24-stigmastadienol, Δ7-stigmastenol, and Δ7-avenasterol.^34^

**Table 1:** Calibration Curves with Sterol Standards Obtained by GC-FID

sterol	range (μg in assay)	calibration equation^a^	linear correlation coefficient
campesterol	24.5–196	*y* = 0.0047*x* +0.0137	0.9961
sitostanol	10.1–303.9	*y* = 0.0046*x* +0.0131	0.9994
stigmasterol	2.9–15.1	*y* = 0.0060*x* −0.0029	0.9931
β-sitosterol^b^	246–1928.6	*y* = 0.0044*x* + 0.0021	0.9988
	0.6–29.4	*y* = 0.0045*x* −0.0004	0.9990

a *y* = sterol area/internal standard area and *x* = micrograms of sterol.

b As a consequence of the large differences in the contents present in the samples, two sets of calibration curves at different concentration ranges were used.

### Statistical Analysis

A one-way analysis of variance (ANOVA), followed by Tukey’s post hoc test, was applied to determine statistically significant differences (*p* < 0.05) in BF contents and bioaccessibility for the same compound (individual or total PS) between different digestion conditions (A, B, or C). This test was also used to evaluate statistically significant differences (*p* < 0.05) in the bioaccessibility between individual PS for the same digestion condition or sample preparation. Additionally, to evaluate statistically significant differences (*p* < 0.05) in BF contents and bioaccessibility for the same compound (individual or total PS) between different sample preparations, a *t*-test was applied. Statistical software Statgraphics Plus 5.1 (Statpoint Technologies Inc. Warrenton, VA) was used throughout.

## Results and Discussion

### Human Oral Phase

The parameters of the increase of the weight of the oral bolus, number of chewing cycles, chewing time, chewing frequency, and oral bolus consistency were determined in the subjects who participated in the study (Table 2). The increase of the bolus ranged from 8.3 to 74.4% (0.08:1 to 0.7:1 (w/w) food/saliva ratio) with a positive correlation (*R*^2^ = 0.66) between the increase of the bolus and chewing time. In the study by Jourdren et al.,^22^ eight subjects (four females and four males) aged between 24 and 37 years old chewed WWB, and an average increase of the oral bolus to about 1:1 (w/w) food/saliva ratio or 100% increase of the bolus was reported. A lower percentage (21–22%) when chewing dried white wheat bread has been indicated.^25^ It should be noted that a 1:1 (w/w) food/saliva ratio is advised by the oral phase in the INFOGEST protocol, as this proportion allows the formation of a swallowable bolus for almost all types of food.^15^

**Table 2:** Chewing Parameters and Bolus Consistency After Human Chewing of Wholemeal Rye Bread by Various Subjects

subject	WRB (g)	oral bolus (g)	increase of the bolus (%)^a^	number of chewing cycles	chewing time (s)	chewing frequency^b^ (s^–1^)	bolus consistency
S1	5.10 ± 0.07	5.52 ± 0.25	8.29 ± 6.33	27.67 ± 2.52	21.38 ± 3.22	1.29 ± 0.28	dense and poorly hydrated
S2	5.16 ± 0.13	8.99 ± 0.10	74.41 ± 4.74	40 ± 0	35.70 ± 1.23	1.12 ± 0.04	like tomato or mustard paste
S3	5.08 ± 0.03	8.60 ± 0.55	69.40 ± 11.72	48.67 ± 5.03	35.63 ± 3.66	1.37 ± 0.09	dense tomato paste
S4	5.09 ± 0.08	8.10 ± 0.45	59.17 ± 8.38	47 ± 1	35.80 ± 3.35	1.31 ± 0.15	dense tomato paste
S5	5.20 ± 0.04	7.18 ± 0.23	38.13 ± 5.25	26.67 ±1.53	23.78 ± 6.46	1.12 ± 0.31	dense and poorly hydrated
S6	5.34 ± 0.10	7.13 ± 0.35	33.68 ± 6.33	31.67 ± 2.52	23.00 ± 2.83	1.38 ± 0.08	dense tomato paste

a Data expressed as mean ± standard deviation (*n* = 3); WRB: wholemeal rye bread; calculated as: (oral bolus-WRB) *x* 100/WRB.

b Calculated as: the number of chewing cycles/chewing time.

In our study, the number of chewing cycles varied between 27 and 49, and the chewing time varied between 21.4 and 35.8 s (Table 2). Similar values of chewing time (27–28 s) for dried white wheat bread were reported by Hoebler et al.^25^ (24 healthy volunteers between 20–55 years of age). Another study performed with 16 healthy subjects (eight females and eight males aged between 16–60 years) indicated a number of cycles and chewing time between 28 and 46 and 17 and 30 s, respectively, during the oral digestion of other cereal products (toast and cake).^37^ However, our results are higher than those reported in a study performed with 14 healthy subjects (10 females and 4 males aged between 24 and 37) masticating WWB (13–20 number of chewing cycles and 11.0–16.7 s for chewing time) and white bread (11–17 number of cycles and 11.8–17.8 s for chewing time),^23^ as well as in the study carried out by Motoi et al.^24^ with white bread (25 cycles and chewing time of 15.3 s) in 12 healthy subjects (seven females and five males aged between 20–29). In addition, the larger number of cycles and mastication time for the WRB in our study can be due to the fact that larger times are needed to obtain an adequate particle size of the oral bolus to be swallowed. In this regard, Nordlund et al.^21^ obtained greater particle size (5.1 *vs* 3.4 mm, respectively) after an oral phase of commercial WRB and WWB (60% wholemeal wheat flour and 40% wheat flour) chewed by four subjects during a fixed chewing time (15 s). Moreover, a positive correlation is observed between the number of chewing cycles and chewing time (*R*^2^ = 0.73) in our study, as indicated by Motoi et al.^24^ in white bread (*R*^2^ = 0.91).

Additionally, the chewing frequency ranged between 1.12 and 1.38 s^–1^ in our work (Table 2). These values are within those indicated by Aleixandre et al.^23^ (0.97 to 1.47 s^–1^) for WWB. No correlation has been found for chewing frequency with any of the above parameters. In any case, even with similar dental status, other physiological factors such as muscle masticatory efficiency and saliva α-amylase activity affect the assessed parameters (increase of the bolus, number of chewing cycles, chewing time, chewing frequency, and oral bolus consistency), explaining the variability observed between subjects since these attributes are shown to be subject-dependent.^15,38^

Subject number S2 achieved a food/saliva ratio closest to 1:1 (w/w) or 100% increase of the bolus (Table 2) and the bolus consistency (not thicker than tomato or mustard paste) indicated by the INFOGEST model^15^ and, hence, this volunteer was selected for the *in vitro* methods assayed (A, B, C).

### Activity of the Human α-Amylase

In the selected subject (S2, 41 years old), the salivary α-amylase activity was 245.3 ± 16.9 U/mL saliva. This value is within the range indicated by other authors: for 112 subjects (equal number of males and females) divided into two groups: 18–25 years (91.9–249.6 U/mL saliva) and 40–60 years (76.2–159.1 U/mL saliva);^33^ for eight healthy subjects (four males and four females, aged from 24 to 37 years) (38–400 U/mL saliva);^22^ and one nonsmoker volunteer without indicating the age (352 ± 41 U/mL saliva).^39^ Moreover, our value is higher than that reported for 13 subjects (eight males and five females) aged between 26 and 52 years (45.6 ± 19.8 U/mL saliva).^40^ Differences in physiological status and age of the subjects can cause a high variability and a wide range in the α-amylase activity.^22^ The flow rate in the saliva of the selected subject was 0.38 ± 0.03 mL saliva/min, which agreed with the value (0.30–0.53 mL/min) indicated by Gavião et al.^37^ and Pedersen et al.^41^

According to the INFOGEST protocol, 75 U α-amylase/mL oral digesta is required in the oral phase. In our case, based on the obtained α-amylase activity (245.3 ± 16.9 U/mL saliva) of the subject S2, the α-amylase activity achieved in the *in vivo* oral phase of the digestion was close to this value (98 ± 6.8 U/mL oral digesta).

### Influence of Enzymes of Lipid Metabolism on Bioaccessibility of Plant Sterols

The initial model INFOGEST^12^ was updated with the addition of GL, a key enzyme for lipid metabolism, thus relevant for their digestion.^15^ It has been reported that the GL produces hydrolysis of triacylglycerides (10–30%), which results in the formation of FFA and MAG, which can act as emulsifiers, increasing the solubilization of lipidic compounds, such as sterols.^14,16^ In addition, these lipolysis products, together with bile salts, promote the formation of mixed micelles, which are necessary for the effective absorption of dietary lipids.^42^ The CE is not only a key enzyme for lipid metabolism present in *in vivo* digestion and is able to hydrolyze esterified sterols, but also it is known that it can act on tri-, di-, and MAG and phospholipids.^14^

For the first time, this study evaluates the effect of the addition of both enzymes of lipid metabolism (only GL at 60 U/mL or combined with CE at 0.075 U/mL) in a solid food with a high fiber content (13.7 ± 1.5/100 g WRB) such as 100% WRB enriched with PS.

The PS identified in the bread and the BF are indicated in Tables 3 and 4. Campesterol, campestanol, stigmasterol, β-sitosterol, sitostanol, Δ5-avenasterol, and Δ7-avenasterol come from the whole rye grain^34,36^ and thus from the flour used to make the bread.^34^ In addition, these sterols have also been detected in the PS source ingredient used to enrich the bread as reported in a similar ingredient.^43^

**Table 3:** Sterol Content in Bioaccessible Fractions (mg sterol/100 g bread) and Bioaccessibility (%) with the Human Oral Phase and Different Gastrointestinal Digestion Conditions^a^

		condition A^12^		condition B^14^ with gastric lipase		condition C^25^ with gastric lipase and cholesterol esterase	
sterols	wholemeal rye bread	BF	BA	BF	BA	BF	BA
campesterol	156.20 ± 7.20	38.51 ± 1.54 a	24.65 ± 0.98 a, x	30.04 ± 1.50 b	19.23 ± 0.96 b, wxy	27.74 ± 1.40 c	17.76 ± 0.89 c, v
campestanol	23.34 ± 2.08	6.95 ± 0.35 a	29.78 ± 1.05 a, w	5.37 ± 0.59 b	23.00 ± 2.54 b, wx	4.97 ± 0.44 b	21.31 ± 1.89 b, wx
stigmasterol	9.29 ± 0.59	2.37 ± 0.17 a	25.46 ± 1.79 a, wx	1.74 ± 0.22 b	18.75 ± 2.34 b, wxy	1.78 ± 0.06 b	19.13 ± 0.68 b, xy
β-sitosterol	1722.87 ± 72.91	404.15 ± 15.66 a	23.46 ± 0.91 a, x	312.76 ± 13.47 b	18.15 ± 0.78 b, wy	291.18 ± 13.76 c	16.90 ± 0.89 b, y
sitostanol	227.28 ± 9.48	54.90 ± 2.18 a	24.16 ± 0.96 a, x	43.92 ± 1.72 b	19.32 ± 0.76 b, wxy	39.00 ± 2.23 c	17.16 ± 0.98 c, y
Δ5-avenasterol	15.26 ± 1.10	3.78 ± 0.23 a	24.77 ± 1.50 a, x	3.57 ± 0.42 ab	23.37 ± 2.75 ab, w	3.14 ± 0.14 b	20.56 ± 0.90 b, wx
Δ5,24-stigmastadienol	5.11 ± 0.74	0.91 ± 0.24 a	17.84 ± 4.63 a, y	0.74 ± 0.28 a	14.57 ± 5.52 a, y	0.69 ± 0.13 a	13.47 ± 2.58 a, z
Δ7-stigmastenol	13.68 ± 1.15	4.09 ± 0.25 a	29.90 ± 1.84 a, w	3.03 ± 0.41 b	22.16 ± 3.03 b, wx	3.01 ± 0.21 b	22.02 ± 1.50 b, w
Δ7-avenasterol	7.64 ± 1.00	3.85 ± 0.20 a	50.38 ± 2.56 a, v	3.08 ± 0.26 b	40.32 ± 3.37 b, v	1.96 ± 0.13 c	25.61 ± 1.65 c, v
total PS	2180.37 ± 90.43	517.97 ± 21.49 a	23.76 ± 0.99 a	403.10 ± 17.00 b	18.49 ± 0.78 b	373.47 ± 17.98 c	17.13 ± 0.82 c

a Sterol content in the bioaccessible fraction (BF) and bioaccessibility (BA) (sterol content in bioaccessible fraction × 100/sterol content in bread) are expressed as mean ± standard deviation (*n* = 6). PS: plant sterols. Different lowercase letters indicate statistically significant differences (*p* < 0.05) for the same sterol between the different methods (BF or BA) (a–c) or between sterols for the same method (BA only) (v–z).

**Table 4:** Sterol Content in Bioaccessible Fractions (mg sterol/100 g bread) and Bioaccessibility (%) Using the *In Vitro* Oral Phase (with α-Amylase) and Gastrointestinal Condition C^a^

		fresh wholemeal rye bread		partially dried and milled wholemeal rye bread	
sterols	wholemeal rye bread	BF	BA	BF	BA
campesterol	159.47 ± 6.68	21.15 ± 3.08 a	13.26 ± 1.93 a, y	32.90 ± 1.66 b	20.63 ± 1.04 b, yz
campestanol	17.61 ± 2.51	4.28 ± 0.60 a	24.30 ± 3.39 a, v	6.38 ± 0.31 b	36.25 ± 1.76 b, v
stigmasterol	8.76 ± 0.80	1.67 ± 0.28 a	19.08 ± 3.17 a, vw	3.08 ± 0.21 b	35.11 ± 2.39 b, v
β-sitosterol	1815.75 ± 116.78	226.88 ± 34.12 a	12.50 ± 1.88 a, y	353.68 ± 18.33 b	19.48 ± 1.01 b, z
sitostanol	225.09 ± 30.95	30.41 ± 6.05 a	13.51 ± 2.69 a, xy	50.78 ± 2.17 b	22.56 ± 0.96 b, xy
Δ5-avenasterol	20.56 ± 2.64	2.63 ± 0.65 a	12.80 ± 3.14 a, y	3.92 ± 0.20 b	19.07 ± 0.99 b, z
Δ5,24-stigmastadienol	4.04 ± 0.35	0.62 ± 0.14 a	15.39 ± 3.59 a, vwx	1.23 ± 0.21 b	30.34 ± 5.10 b, w
Δ7-stigmastenol	15.58 ± 0.91	2.50 ± 0.35 a	16.04 ± 2.25 a, wxy	3.79 ± 0.36 b	24.31 ± 2.33 b, x
Δ7-avenasterol	10.31 ± 1.58	2.46 ± 0.46 a	23.91 ± 4.48 a, v	3.30 ± 0.19 b	32.07 ± 1.85 b, w
total PS	2277.79 ± 158.62	292.46 ± 44.77 a	12.84 ± 1.97 a	459.06 ± 22.98 b	20.15 ± 1.01 b

a Sterol content in the bioaccessible fraction (BF) and bioaccessibility (BA) (sterol content in bioaccessible fraction × 100/sterol content in bread) are expressed as mean ± standard deviation (*n* = 6). PS: plant sterol. Different lowercase letters indicate statistically significant differences (*p* < 0.05) for the same sterol between the different methods (BF or BA) (a, b) or between sterols for the same method (BA only) (v–z).

In our study, we have also identified and quantified Δ7-stigmastenol and Δ5,24-stigmastadienol (Tables 3 and 4), which have also been detected in milling fractions of rye grain although no content values are reported.^36^ However, Δ7-stigmastenol has been indicated as an artifact of β-sitosterol due to the use of high-temperature conditions and alkaline media, such as in the saponification process carried out with saturated KOH at 80 °C,^44^ and Δ5,24-stigmastadienol can be derived from the isomerization of Δ5-avenasterol.^36^ Moreover, Piironen et al.^34^ reported the combined quantification of other nonidentified sterols in the whole rye grain and flour.

The PS content in the BF (mg/100 g) and their bioaccessibility in WRB are shown in Table 3 for the human oral phase and digestion conditions (A, B, and C) (see the Oral–Gastrointestinal Simulated Digestion section). The total PS content in the BF varied between 373.5 and 518 mg/100 g, being higher for method A, followed by methods B and C, with statistically significant differences (*p* < 0.05) between the three methods. However, the difference between methods B and C is considered to be of little relevance, when compared to method A, and the same trend occurred in the bioaccessibility values of total PS (A: 23.8%; B: 18.5%; and C: 17.1%). Regarding individual PS, their contents in the BF were higher in method A (0.9–404.2 mg/100 g), followed by methods B (0.7–312.8 mg/100 g) and C (0.7–291.2 mg/100 g), as it was observed for bioaccessibility values. The most bioaccessible PS was Δ7-avenasterol (25.6–50.4%), and Δ5,24-stigmastadienol showed the lowest bioaccessibility (13.5–17.8%). Statistically significant differences (*p* < 0.05) were detected between method A *vs* B and C in the contents of individual PS in the BF and their bioaccessibility, except for Δ5,24-stigmastadienol and Δ5-avenasterol (Table 3). When comparing method B *vs* C, statistically significant differences (*p* < 0.05) were observed in the content of BF and bioaccessibility for campesterol, β-sitosterol (only in the BF content), sitostanol, and Δ7-avenasterol but not for campestanol, stigmasterol, Δ5-avenasterol, and Δ7-stigmastenol.

So far, no studies have determined the bioaccessibility of PS in WRB (PS-enriched or not). In cereals, the bioaccessibility of steryl ferulates in cargo rice and rice bran, corn bran, and wheat bran has been determined by Mandak et al.^26^ using an oral phase with α-amylase (without indicating enzyme activity), gastric phase without GL, and duodenal stage with lipase and CE provided by the pancreatin added. After simulated digestion, the bioaccessibility of steryl ferulates ranged between 0.0 and 1.5%. The decrease of steryl ferulates after digestion can be explained by the hydrolysis of steryl ferulates and steryl fatty acid esters, which are good substrates for CE. These authors in a subsequent study^27^ evaluated the effect of simulated digestion of integral wheat flour and combined with white wheat flour, and their respective breads, obtaining bioaccessibility of steryl ferulates of 0.03–0.09% for both flours and 0.01–0.25% for breads. The difference in bioaccessibility between flour and bread samples may be due to the action of endogenous lipase present in flour, which is activated during digestion. However, the process of baking at high temperatures may denature it, inhibiting its action. Our results cannot be directly compared to these two studies since only the steryl ferulate bioaccessibility was assessed and a different *in vitro* digestion method was applied.

In another study carried out with PS-enriched granola bars (oat cereal) using different formulations (crude PS + empty nanoporous starch aerogels, crude PS + pregel starch, and PS-nanoporous starch aerogels), the bioaccessibility of total PS was evaluated.^28^ Granola bars were formulated with different percentages of fat from canola oil (regular-fat, low-fat, and nonfat with 24, 7, and 0/100 g sample, respectively, and 1 g of PS content added to all formulations). The INFOGEST digestion model is used with modifications (grounding the bars to simulate mastication in the oral phase and using fungal lipase instead of GL from the gastric rabbit extract). The fortification with PS-nanoporous starch aerogels obtained the highest bioaccessibility, being 91.8% for regular-fat granola bar, followed by low-fat granola bar (88.2%) (no statistically significant differences (*p* < 0.05) between them) and nonfat granola bar (52.7%). Higher bioaccessibility is contributed to the fat content due to a better micellarization. The bioaccessibility obtained in our study under similar digestion conditions (method B with addition of lipase, although not fungal origin) is 18.5%, being lower possibly due to the differences in the methodology, food matrix (granola bars *vs* WRB), and content of lipids (24, 7, or 0/100 g granola bars *vs* 3.2/100 g WRB).

Although no cholesterol was identified in the WRB as expected, pancreatin, bile salts, and RGE providing GL are digestion reagents that can provide cholesterol preformed in micelles during the digestion process.^30^ It is indicated that, in addition to the cholesterol provided by the ingredient of milk fat globule membrane (0.63 mg/5 g PS-enriched milk-based fruit beverage), another 2.75 mg came from the pancreatin, bile salts, and RGE (1.7, 1.0, and 0.05 mg of cholesterol/total digesta, respectively), and reagents used in the digestions performed under the same conditions as in our study.^30^ A higher PS content in 5 g of WRB (109.1 *vs* 45.6 mg of PS in the beverage) and a lower content of cholesterol in the digestion (2.75 mg in WRB *vs* 3.38 mg in the beverage) could explain the increase in total PS bioaccessibility detected in WRB *vs* beverage, being 23.8 *vs* 13.7% in method A, 18.5 *vs* 7.4% in method B with GL addition, and 17.1 *vs* 8% in method C with GL combined with CE. The same trend is observed for individual PS, being higher the bioaccessibility for method A in WRB (ranged 17.8–50.4%) *vs* beverage (9.7–19.7%). It has been observed that the use of GL (method B) decreases the bioaccessibility of total and individual PS compared to method A, as seen in Makran et al.,^30^ for the beverage. In the presence of GL, the lipolysis products (FFA and MAG) could increase the incorporation of cholesterol, especially favorable when provided by digestion reagents, into the mixed micelles, thus displacing PS.^14,16^ In this regard, Wilson and Rudel^17^ observed that *in vivo* biliary cholesterol is more easily incorporated because it is in preformed micelles, in opposition to dietary cholesterol or sterols, which need prior emulsification. In addition, the rate of GL lipolysis varies depending on the matrix, being 25% for liquid foods and lower (10%) for solid foods. Liquid test meal provided a better substrate for lipases probably because they are pre-emulsified and stabilized, while in the solid meal, the physicochemical state of the lipids is more heterogeneous, and most of the triacylglycerides have to be emulsified during the digestion.^45^ Therefore, this fact would also justify the higher bioaccessibility of PS in the WRB *vs* the beverage^30^ since the lower GL action in the WRB decreases the incorporation of cholesterol into the micelles, allowing the incorporation of PS.

Regarding the effect of CE (used in method C), a comprehensive review^18^ reported that this enzyme has a supplementary or compensatory effect on lipolytic activity. This effect could increase the products of lipolysis (FFA and MAG), which would favor the incorporation of cholesterol into the mixed micelles, displacing the PS even more than the GL effect. Although there are statistically significant differences (*p* < 0.05) of bioaccessibility for total PS between methods B (addition of GL) and C (addition of GL combined with CE) (18.5 *vs* 17.1%, respectively), they can be considered of no functional relevance. The INFOGEST digestion model incorporating CE (0.1 U/mL) was used to evaluate the effect on the bioaccessibility of PS esters used to enrich soybean oil. The statistically significant (*p* < 0.05) increase in the bioaccessibility of PS (6 *vs* 4% without CE) was attributed to the hydrolysis of the PS esters by the action of CE,^46^ which does not agree with our study, where the PS added are in free form.

### Influence of Oral Phase and Fiber Content on Bioaccessibility of Plant Sterols

Table 4 shows the PS content in the BF (mg/100 g) and their bioaccessibility in fresh or partially dried and milled WRB after an *in vitro* oral phase (SSF and α-amylase). The total PS content in the BF for fresh WRB was 292.5 mg/100 g, being statistically different (*p* < 0.05), and lower than the content obtained by partially dried and milled WRB (459.1 mg/100 g). The same trend was observed in the total PS bioaccessibility (12.8 *vs* 20.2%). Statistically significant differences (*p* < 0.05) exist for all individual PS content in the BF and bioaccessibility between fresh and partially dried and milled WRB (Table 4). The bioaccessibility of PS (total and individual) was lower in the fresh *vs* partially dried and milled WRB probably because the crust has been poorly homogenized in the *in vitro* oral phase, decreasing the accessibility for the digestive enzymes, the release of PS, and thus their incorporation into the mixed micelles. In this regard, a threefold higher variability in BF contents and bioaccessibility values is detected when the fresh WRB is digested compared to the partially dried and milled WRB (15 *vs* 5% of relative standard deviation, respectively). Moreover, different solubility patterns are shown depending on the different sample preparations, i.e., the most bioaccessible PS in the digestion from fresh WRB were campestanol, Δ7-avenasterol, stigmasterol, and Δ5,24-stigmastadienol with no statistically significant differences (*p* < 0.05) between them. Sterols with the lowest bioaccessibility were Δ7-stigmastenol, sitostanol, campesterol, Δ5-avenasterol, and β-sitosterol with no statistically significant differences (*p* < 0.05) between them. When partially dried and milled WRB was digested, the highest bioaccessible sterols were campestanol and stigmasterol (with no statistically significant differences (*p* < 0.05)), while campesterol, β-sitosterol, and Δ5-avenasterol were the sterols with the lowest bioaccessibility (with no statistically significant differences (*p* < 0.05)).

The influence of sterol–fiber interaction has been poorly studied in relation to sterol bioaccessibility and mainly focused on cholesterol. The addition of 3 or 6 g of partially hydrolyzed guar gum fiber/100 g yogurt (cholesterol/fiber ratio of 0.23 or 0.12, respectively) decreased the bioaccessibility of cholesterol by 9 and 23%, respectively, compared to the control (without fiber addition), using a multicompartimental (gastric, duodenal, and small intestinal) dynamic digestion system.^47^ The authors attributed this decrease to depletion flocculation with the fiber, which reduces cholesterol absorption by decreasing its incorporation into the mixed micelles. Different fiber extracts from lemon, grapefruit, and pomegranate, and subproducts of lemon ice cream and tiger nut “horchata” (beverage) are added to 100 g of pork patties (4.5–6.9 g of total fiber, 1.4–6 g of insoluble fiber, 0.012–5.3 g of soluble fiber).^48^ After the *in vitro* digestion of these samples by applying the standardized INFOGEST method, 68–89% total cholesterol is found in the oily phase and between 6–32% in the pellet phase. In contrast, practically, all the cholesterol is present in the oily phase after the digestion of the control samples (without fiber addition). This fact shows that dietary fiber, regardless of its origin and soluble/insoluble ratio, is able to retain cholesterol (hydrophobic interaction) in the pellet phase, decreasing its incorporation into the oily phase, which corresponds to the BF, where it is theoretically available for absorption.

In a previous study by our research group,^49^ the influence of galactooligosaccharides (GOS) (2.5 and 5 g of GOS/250 mL of a PS-enriched milk-based beverage) on sterol bioaccessibility has been studied using a micellar gastrointestinal *in vitro* digestion with α-amylase, without GL but with CE. The presence of GOS at both tested concentrations did not affect the bioaccessibility of total PS (37.2% without GOS, 37.7% (2.5 g), and 37.1% (5 g)), with sterols/GOS ratios of 0.98 and 0.6, respectively. A lower bioaccessibility of total (17.1–23.8%) and individual PS (Table 3) is obtained in our study from PS-enriched WRB. Different conditions in the *in vitro* digestion (enzymes and activities), different matrices, and a lower PS/fiber ratio (0.16 in WRB) could justify the lower bioaccessibility in a matrix rich in fiber such as the WRB (10.3 ± 1.4 g insoluble fiber/100 g WRB; 3.4 ± 0.1 g soluble fiber/100 g WRB) *vs* the milk-based fruit beverage.

The results obtained in the present work provide relevant information about the use of the INFOGEST digestion protocol for evaluating the bioaccessibility of PS in a PS-enriched WRB, especially regarding the effect of the oral phase. The use of the INFOGEST method remains an appropriate and cost-effective methodology for this purpose as it indicates the requirements to be fulfilled in the oral phase (bolus weight increment of 100% or a 1:1 (w/w) food/saliva ratio and consistency not thicker than tomato or mustard paste). Therefore, the selection of subjects, who comply with these requirements for carrying out the human chewing in the *in vitro* digestion, is crucial due to the high discordance observed in this study between different subjects in the increase of the bolus, the number of chewing cycles, chewing time, and frequency and oral bolus consistency. Additionally, the use of the *in vitro* oral phase proposed by INFOGEST (SSF and α-amylase) for the digestion of the WRB provides high variability in the PS bioaccessibility in the fresh sample, compared to the partially dried and milled, due to an ineffective homogenization. The main advantage of the adaptation of the INFOGEST protocol proposed in this study is the use of an *in vivo* oral phase applied to a solid food matrix, which makes it a more physiological situation within an *in vitro* digestion method. However, the use of this *in vivo* oral phase has some limitations, such as a high variability between subjects, making preliminary studies necessary to choose the candidate best suited to the requirements detailed in the INFOGEST digestion. We can conclude that, for solid matrixes, such as bread, enriched with bioactive lipophilic compounds as PS, an oral phase with human chewing and the incorporation of enzymes of lipid metabolism (GL and CE) in the INFOGEST method is proposed when assessing PS bioaccessibility to provide a more physiological approach to the *in vivo* gastrointestinal scenario.

## Abbreviations Used

BF: bioaccessible fraction
CE: cholesterol esterase
FFA: free fatty acids
GC-FID: gas chromatography-flame ionization detector
GL: gastric lipase
GOS: galactooligosaccharides
HMDS: hexamethyldisilazane
IS: internal standard
MAG: monoacylglycerides
PS: plant sterols
RGE: rabbit gastric extract
SGF: simulated gastric fluid
SIF: simulated intestinal fluid
SSF: simulated salivary fluid
TMCS: trimethylchlorosilane
WRB: wholemeal rye bread
WWB: wholemeal wheat bread
